# Recommended characteristics and processes for writing lay summaries of healthcare evidence: a co-created scoping review and consultation exercise

**DOI:** 10.1186/s40900-023-00531-5

**Published:** 2023-12-20

**Authors:** Sareh Zarshenas, JoAnne Mosel, Adora Chui, Samantha Seaton, Hardeep Singh, Sandra Moroz, Tayaba Khan, Sherrie Logan, Heather Colquhoun

**Affiliations:** 1 Department of Occupational Science and Occupational Therapy, University of Toronto, Toronto, ON Canada; 2 Patient Partner, The Strategy for Patient-Oriented Research (SPOR)-Evidence Alliance, Toronto, ON Canada; 3 Institute of Health Policy, Management and Evaluation, University of Toronto, Toronto, ON Canada; 4 Rehabilitation Sciences Institute, Temerty Faculty of Medicine, University of Toronto, 500 University Ave, Toronto, ON Canada; 5 KITE Toronto Rehabilitation Institute-University Health Network, Toronto, ON Canada

**Keywords:** Lay summary, Plain language summary, Healthcare evidence, Patient partners, Public partners, Cocreation, Characteristics, Creation processes

## Abstract

**Background:**

Lay summaries (LSs) of scientific evidence are critical to sharing research with non-specialist audiences. This scoping review with a consultation exercise aimed to (1) Describe features of the available LS resources; (2) Summarize recommended LS characteristics and content; (3) Outline recommended processes to write a LS; and (4) Obtain stakeholder perspectives on LS characteristics and writing processes.

**Methods:**

This project was a patient and public partner (PPP)-initiated topic co-led by a PPP and a researcher. The team was supported by three additional PPPs and four researchers. A search of peer-reviewed (Ovid MEDLINE, Scopus, Embase, Cochrane libraries, CINAHL, PsycINFO, ERIC and PubMed data bases) and grey literature was conducted using the Joanna Briggs Institute Methodological Guidance for Scoping Reviews to include any resource that described LS characteristics and writing processes. Two reviewers screened and extracted all resources. Resource descriptions and characteristics were organized by frequency, and processes were inductively analyzed. Nine patient and public partners and researchers participated in three consultation exercise sessions to contextualize the review findings.

**Results:**

Of the identified 80 resources, 99% described characteristics of a LS and 13% described processes for writing a LS. About half (51%) of the resources were published in the last two years. The most recommended characteristics were to avoid jargon (78%) and long or complex sentences (60%). The most frequently suggested LS content to include was study findings (79%). The key steps in writing a LS were doing pre-work, preparing for the target audience, writing, reviewing, finalizing, and disseminating knowledge. Consultation exercise participants prioritized some LS characteristics differently compared to the literature and found many characteristics oversimplistic. Consultation exercise participants generally supported the writing processes found in the literature but suggested some refinements.

**Conclusions:**

Writing LSs is potentially a growing area, however, efforts are needed to enhance our understanding of important LS characteristics, create resources with and for PPPs, and develop optimal writing processes.

**Supplementary Information:**

The online version contains supplementary material available at 10.1186/s40900-023-00531-5.

## Abstract (plain language summary)

This study was suggested by a patient partner to place attention on the role patient and public partners (PPPs) could play in developing lay summaries. A lay summary (LS) is a summary of a research project written for members of the public, including patients. A lot of information is written about recommendations for LSs, but none of it has been summarized. This study: (1) Pulled together and summarized all existing resources that made recommendations on features of LSs and/or the steps for writing them; and (2) Conducted meetings with people interested in LSs (PPPs and researchers) to gather their perspectives on this summary of resources. The study engaged PPPs in all aspects, including co-leadership. We found 80 resources on LSs. Almost all (95%) of the resources were written by researchers for researchers, with only 18% involving PPPs. The most common recommendations were to avoid jargon (78%) and remove unnecessary and complex words (60%). Only 13% of the resources had information about the steps for writing a LS. People in our meetings did not always agree with the recommended LS characteristics and found them overly simplistic. They felt that identifying and writing for the intended audience of the LS was important, every study should have a LS, PPPs should have the opportunity to be involved, and greater attention should be paid to the steps involved in writing a LS. Lay summary development is a complex, multistep process requiring the inclusion of PPPs for their irreplaceable perspectives and contributions.

**Supplementary Information:**

The online version contains supplementary material available at 10.1186/s40900-023-00531-5.

## Introduction

Lay summaries (LSs) are a critical knowledge translation strategy to communicate healthcare research evidence to patient and public partners (PPPs) [1, 2]. By bridging gaps between research evidence and patient and public comprehension, LSs can facilitate more meaningful conversations about healthcare research and its implications, potentially resulting in more informed healthcare decision-making [3, 4]. As defined by the National Institute for Health Research (NIHR) a lay summary is “a research project or a research proposal summary that has been written for members of the public, rather than researchers or professionals” [5]. LSs should avoid jargon, explain technical terms, and use plain language [5].

There is a growing interest and effort in publishing LSs of healthcare evidence. Various institutions and knowledge brokers, such as agencies (e.g., Canadian Frailty Network [6], MS Canada [7]), journals, and publishers (e.g., Taylor and Francis [8], Wiley [9]), offer guidance for writing LSs. The Cochrane Collaboration has developed plain language summary guidance specifically for Cochrane reviews, which includes a template and plain language summary preparation steps [10]. In 2018, the European Clinical Trial Regulation 536/2014 (EU-CTR) launched the second version of its guidance on producing clinical trial summaries for lay persons [11]. This document includes a template to help authors write LSs for clinical trials and contains a framework that describes specific LS characteristics across seven over-arching principles [i.e., general principles (e.g., develop the summary for a general public audience), health literacy principles and writing style (e.g., text should be proper for people with a low to average level of literacy), readability and use of plain language (e.g., sentences should be kept short and concise), numeracy (e.g., numerical data should be easily understandable), visuals (e.g., using well-chosen and clearly designed visual aids), language (e.g., using a local language), and communication of results with participants (e.g., presenting results to patients and receive their feedback)] [11].

The significance of involving PPPs in the development, implementation, and dissemination of health-related evidence is increasingly being acknowledged, particularly in the realm of knowledge translation strategies [4, 12, 13]. Despite growing interest in LS guidance, a wide range of guidance documents are dispersed across many health and healthcare sectors with uncertainty on the optimal LS characteristics and processes for writing LSs [6–10]. These challenges are particularly pronounced for PPPs seeking to participate in the LS writing processes [14]. Although the primary aim of a LS is to facilitate access to scientific evidence, role of PPPs in the writing of LSs has been less well-established [3, 15–17].

The aim of this study was to conduct a scoping review of existing LS guidance specific to recommended LS characteristics (i.e., what LSs should look like) and writing processes (i.e., how best to write a LS). We further aimed to conduct a consultation exercise with a range of PPPs and researchers interested in LSs to obtain knowledge user perspectives on the results of the scoping review.

Our specific objectives were to:

1. Describe the features of available LS resources regarding source type (i.e., peer-reviewed, grey literature), country, publication year, focus (i.e., LS characteristics, LS writing process, both), PPP involvement in the guidance creation (i.e., yes/no), specified target audience, and specific context (e.g., reviews, clinical trials) or condition (e.g., autism).
2. Summarize recommended LS characteristics and content using an adapted version of the EU-CTR framework principles (i.e., health literacy principles and writing style, readability and use of plain language, numeracy, visuals, and language) [11].
3. Summarize recommended processes for writing a LS.
4. Obtain stakeholder perspectives (i.e., PPPs and researchers) on LS characteristics and writing processes (i.e., results of objectives 2 and 3).

## Methods

### Study design

A scoping review with a consultation exercise was conducted using the Joanna Briggs Institute Methodological Guidance for Scoping Reviews [18] and was reported by employing the Preferred Reporting Items for Systematic Reviews and Meta-Analyses Extension for Scoping Reviews-PRISMA- ScR) [19]. A protocol of this project was published with detailed information on methodology [20]. We conducted electronic searches on eight databases, including Ovid MEDLINE, Scopus, Embase, Cochrane libraries, CINAHL, PsycINFO, ERIC, and PubMed (see online supplemental file 2 for an example of the Ovid MEDLINE search strategy). Additionally, grey literature was searched to ensure the inclusion of relevant health documents from governmental and non-governmental agencies, organizations, and community associations. Our grey literature search strategy involved (a) following The Canadian Agency for Drugs and Technologies in Health guidance for health-related grey literature searches, (b) searching for pertinent documents on the first 10 pages of Google, and (c) seeking suggestions from research team members for additional resources. The grey literature search limited to English language countries, including Canada, the USA, the UK, and Australia. Furthermore, we supplemented our research by manually inspecting the reference lists of selected articles to identify related documents that might not have been captured in the aforementioned search strategies (Additional file 1: Table S1, databases search strategy).

The consultation exercise was approved by the research ethics board of the University of Toronto (REB approval number: 43453). Participants submitted a signed written consent in compliance with the approved Research Ethics Board (REB) requirements prior to engaging in the consultation exercise.

### Patient and public partners involvement

This project was conceived and initiated by a PPP (JM) and funded by the Strategy for Patient-Oriented Research-Evidence Alliance (SPOR-EA) [21]. The SPOR-EA is jointly funded by the Canadian Institutes of Health Research (CIHR) under the SPOR initiative, and it includes 41 partners from public and not-for-profit sectors across Canada [22]. In 2021 the SPOR Evidence Alliance initiated a funding opportunity in which PPPs submitted research questions to be prioritized for funding and to guide the research efforts of the Alliance. The topic of LSs was submitted by our PPP based on years of LS advocacy work and the need to increase access to high quality LSs of scientific evidence. The original proposal was to synthesize the literature that supports PPPs in writing LSs of scientific evidence. The SPOR-EA acknowledged the significance of this topic to PPPs and its potential impact and funded this work; however, an initial scoping search showed a scarcity of existing literature on this topic [13]. Thus, our team embarked on an iterative process of refining the original intent into the objectives presented here.

The project was co-led by a PPP (JM) and a researcher (HC). The team included four additional researchers (SZ, AC, HS, SS), and three PPPs (SM, TK, SL). PPPs were full members of the research team as they provided input on all project processes. Monthly team meetings occurred throughout the project with weekly meetings between the two co-leads at various time points in which the PPP co-lead input was critical (e.g., planning the consultation exercise). To facilitate PPP engagement, the integrated Knowledge Translation (iKT) approach was applied to this study [23]. Detailed information regarding applying the iKT approach was provided in the protocol [20]. PPPs engaged in learning opportunities related to screening citations and one PPP (SL) was the second screener/extractor. Our team created an infographic of the study roadmap that serves as a valuable tool for improving communication and comprehension of the scoping review process (Additional file 2: Fig. S2). To provide detailed information on our PPP involvement, we used the short form of the Guidance for Reporting Involvement of Patient and the Public (GRIPP)-2 [24] (Table 1).

**Table 1:** Guidance for reporting involvement of patients and the public (GRIPP)2-short form

1. Aim: Report the aim of PPI* in the study
This project was unique in that the patient and public partner (PPP) co-lead was the person who initiated the study. This was achieved through a PPP submission to the Strategy for Patient-Oriented Research (SPOR) Evidence Alliance^**^ in response to an identified gap in writing lay summaries by researchers and PPPs. In addition to the PPP co-lead, we had three other patient partners as full research team members. The aim for integrating these PPPs into the study was to shape the scoping review, support the collection of data that was meaningful to PPPs, provide opportunities for learning about the conduct of knowledge syntheses, integrate PPP perspectives in the completion of a consultation exercise as part of the scoping review, and to ensure the outputs and resources created met the needs and preferences of PPPs
*2. Methods: Provide a clear description of the methods used for PPI in the study*
We used an integrated Knowledge Translation (iKT) approach to ensure PPPs had the opportunity to participate in all study processes. The iKT approach stresses the equitable involvement of stakeholders in research and ensures the engagement of the primary knowledge users (i.e., PPPs) across all steps of this project, as per their interest. This approach included PPP involvement in identifying the research questions, supporting the writing of the protocol, collecting data, interpreting findings, and developing the final outputs. Key principles were co-created for how our team would collaborate to ensure clear communication, maximize contributions, and provide a safe space for all team members, including PPPs. An infographic, which was regularly discussed at team meetings, was created for each project step to facilitate understanding of the review steps. Monthly team meetings occurred to review project progress and receive feedback. Additional regular meetings took place between the PP and researcher co-lead. To contextualize the scoping review findings, a consultation exercise was conducted in which all PPPs engaged in planning and analysis and one PPP took on a co-lead role during the exercise
*3. Study results: Report the results of PPI in the study, including both positive and negative outcomes*
PPPs provided valuable feedback on the study protocol including responses to journal reviewers, drafts of the proposal submitted to the research ethics board, the data extraction guide, the detailed plans for the consultation exercise, the scoping review manuscript, and the study lay summary. This was achieved through both written and verbal feedback
Researchers provided training to PPPs to use Covidence (literature review screening software) and were engaged in screening peer-reviewed resources. PPPs were also provided with some samples of grey literature to become familiar with various types of literature and specific processes of screening for each type. One PPP was the second screener and extractor for the review, completing this work for the peer-reviewed and grey literature. All four PPPs were involved in the discussion of scoping review results during team meetings and were directly involved in the analysis phase for the consultation exercise. Subsequently, they discussed the potential impact of the findings on different stakeholders, researchers, and the public. They also suggested different platforms to disseminate the results. PPPs reviewed and edited abstracts submitted to scientific conferences and participated in creating posters and presentations. PPPs provided feedback on the final manuscript
*4. Discussion and conclusions: Comment on the extent to which PPI influenced the study overall. Describe positive and negative effects*
The PPPs contributed to identifying patient experiences, needs, priorities, and values and conceptualizing the research problem from PPPs’ perspectives. Their involvement added credibility, meaning, and insight to the study and its findings. Their critical perspectives were particularly constructive in discussing the results of the consultation exercise. The PPP co-lead ensured that the research team remained centred on the project purpose and were aware of critical PPP perspectives as they related to lay summaries. PPPs co-designed the consultation exercise and directly engaged in analyses of the sessions
Project progress was at times slowed by the training and informational needs of the PPPs. Having a PPP co-lead was a beneficial aspect of the project and resulted in a thoughtful approach but also required more time in discussion and collaboration. Engaging in a fulsome co-leadership model was at times challenging given the differences in perspectives and the funding model of reimbursing a PPP as an honorarium. This limited time available for true co-leadership and likely reduced its potential impact. Greater effort should have been spent initially to better establish the co-leadership model from the PPP co-lead perspective
*5. Reflections/critical perspective: Comment critically on the study, reflecting on the things that went well and those that did not, so others can learn from this experience*
The project was a learning experience for all team members. It provided an excellent opportunity for PPPs and researchers to engage in collaborative communication, the development of safe spaces for everyone and learning more about the positive nature of PPP initiated and co-led research
PPPs were able to further their understanding of the research process and researchers were able to further their understanding of PPP perspectives and approaches to PPP engagement, particularly for PPP co-led projects. All team members reported that team processes allowed for reflection on assumptions related to lay summaries
Areas for improvement included having the researcher co-lead be less eager to get the project started and spend more time initially on framing the project together with the PPP co-lead and having more robust engagement of the PPP co-lead for initial steps including search strategy review and protocol development. Efforts were made to include PPP training on every aspect of the review but in the interests of time and review progress, it might have been better to have PPPs prioritize review steps of greatest interest. This could have enhanced these experiences for PPPs. Due to budget limitations, only one PPP had the full experience of study screening and extraction. Different budget considerations might have allowed for additional PPP involvement. A significant learning was to prioritize time initially on expectations of the PPP co-lead model including desired approaches to communication and collaboration

*PPI Patient and public involvement

**SPOR EA is a pan-Canadian Network of 300 + members that promotes best practice in the use of evidence for practice and policy

### Eligibility criteria

Peer-reviewed articles and grey literature documents were included in the scoping review if they: (a) pertained to healthcare, (b) described guidance, recommendations, strategies, or suggestions for LS characteristics and/or writing processes. Non-full text and non-English language resources were excluded due to the limitation of human and material resources required for searching and data extraction process. Resources were not restricted by publication year, context (e.g., review), or health conditions (e.g., autism). Studies that examined different types of LSs, investigated perspectives of knowledge users on LSs, or explored the effects of patient involvement in writing LSs were excluded since these studies do not typically provide specific recommendations for LS characteristics or writing processes. Similarly, studies specific to health literacy were not included as they did not directly align with our central question. Conference abstracts were also omitted due to their limited level of detail. To draw attention to the impact of PPP healthcare decision-making and LSs, we excluded resources specific to guidance on writing manuscripts, summaries for trial participants, animal studies, the needs of policy makers, and increasing PPPs understanding of research in general.

### Sources of data, search strategies and data collection

A search strategy was created by SZ in consultation with HC and an information specialist at the University of Toronto [20]. To enhance the comprehensiveness of the search strategy, Peer-Review of Electronic Search Strategies (PRESS) criteria [25] were used. Peer-reviewed articles were searched electronically using the following eight databases: Ovid MEDLINE, Scopus, Embase, Cochrane libraries, CINAHL, PsycINFO, ERIC, and PubMed [20] and updated on May 30th, 2022. Additionally, grey literature was searched to ensure retrieval of relevant health related LS documents in governmental and non-governmental agencies, organizations, and community associations. To facilitate our grey literature search process, we utilized the Canadian Agency for Drugs and Technologies in Health’s guidance (CADTH’s) “Grey Matters” checklist [26]. We used key search terms such as "lay summary writing," "lay abstract," and "plain language summary guidance" to search relevant websites. We also employed the Google search engine, adhering to the guidance outlined by the University of Toronto library [27]. Lastly, we used the expertise of our team to ensure all resources were explored and searched for any links we found in peer-reviewed articles. We also conducted a thorough hand-search of the reference lists of included resources, ensuring that all potential avenues for available resources were explored.

### Data screening

All peer-reviewed articles obtained from the databases were compiled and duplicates were eliminated using Endnote software. Two reviewers (SZ, SL) independently screened the titles and abstracts of the retrieved articles using the Covidence review platform [28] and evaluated the full texts of the relevant articles based on our inclusion criteria. Grey literature was similarly screened by the same reviewers. To ensure a rigorous screening of the grey literature, each reviewer independently assessed every resource against the pre-determined inclusion criteria. To facilitate the consensus-building process and address any discrepancies or uncertainties, a third person (HC) was present in all consensus meetings.

### Data extraction

An a priori data extraction guide was created in collaboration with the team. To organize the LS characteristics, we adapted the EU-CTR framework [11]. While this framework, as one of the most comprehensive approaches to LS guidance, was a useful starting point, adaptations were necessary to avoid duplication or double-barreled characteristics (e.g., “Removing unnecessary or complex words and/or avoiding long sentences”), procedural characteristics (e.g., "Sponsors should note that there is no limit placed on the size of the lay summary document that will be uploaded as a PDF document”), and characteristics that were being described in our other objectives (e.g., characteristics specific to whether PPPs were involved). The final adapted EU-CTR Framework had 29 characteristics across five principles. Over the course of extraction, nine additional characteristics were added based on their absence from the EU framework and perceived importance by the research team (i.e., indicating a funders’/ sponsors’ name, ensuring availability of LS soon after the study publication, avoiding oversimplifying, mentioning search date/timescale, focusing on the person not the disability, framing language of sentences in the positive way, ensuring LS is indexed in PubMed, spelling out abbreviations, and using inclusive language). There were three characteristics in which we chose to extract an additional level of detail considering the range of presented detail in the resources (i.e., word count, readability test and reading level).

To focus broadly on LSs, we did not extract information from the resources that was specific to a context or condition. For example, a resource that recommended LS characteristics specific to drug trials (e.g., ensure to describe the drug itself) or systematic reviews (e.g., approaches to describing summary tables). To ensure consistency and clarity of the data extraction guide, we conducted multiple pilots of 5% of both peer-reviewed and grey literature resources. The piloting process was completed after reaching 80% agreement between reviewers.

The final extracted variables included: Objective (1) guidance features including source type (i.e., peer-reviewed, grey literature), authors for peer reviewed/organizations for grey literature, country of publication, year, PPP involvement in the guidance creation (i.e., yes/no), target group for the guidance, and if guidance was focused on a specific context (e.g., reviews, clinical trials) or condition (e.g., autism), Objective (2) 29 adapted EU-CTR characteristics (i.e., yes; no), nine additional non-EU-CTR characteristics determined during extraction, three specifications of EU-CTR recommendations (i.e., word count, readability test, reading level), and recommendations for LS content, and Objective (3) the steps for processes to write a LS.

### Data analysis

Descriptive quantitative analysis was used to address objectives 1 and 2 by summarizing and presenting numerical information on the importance of characteristics by rank ordering characteristics in the reviewed literature according to their frequency (n, %). This approach helped in gaining insights into the most prevalent and therefore noteworthy characteristics within the scope of the study.

For the LS writing processes, all recommended steps were summarized and organized in a Microsoft Excel file. We then documented the sub-components of each step, allowing us to create a summary of the processes while still describing each one individually.

### Consultation exercise

Anticipating limited PPP inclusion in the literature, a consultation exercise was conducted. The overall aim of the consultation exercise was to engage a group of PPPs and researchers in contextualizing the scoping review results related to (1) the recommended LS characteristics; and (2) the suggested LS writing processes. These two review areas were prioritized for the consultation exercise due to their relevance to our team PPPs. The consultation exercise participants inclusion criteria included: (1) Interest in the concept of LSs and/or experience in writing/using LSs for health decision making; and (2) Fluency in English. The planned PPP-to-researcher participant ratio was 3:1 to prioritize PPP input while recognizing that the co-creation of LSs with PPPs and researchers is likely good practice. This ratio was a decision made by the entire research team, including our PPPs. They were recruited using a purposeful sampling approach by distributing the study flyer through the SPOR-EA network in Canada. It should be noted that none of the participants of the consultation exercise were members of the research team, including research team members who were PPPs.

Using the Nominal Group Technique (NGT) steps as a guide to design our consultation exercise activity [29] which is explained comprehensively in the study protocol [20], three sessions, each two hours long, were held on Zoom over a five-week period in February and March 2023. All sessions were co-facilitated by the lead researcher (HC) and a patient partner (SL), and all members of the research team attended as observers and note takers. Two weeks prior to the first session, participants were sent a set of materials that included a consent form, participants’ and research team’s bios, an agenda, and a list of LS characteristics with definitions and examples. Participants submitted a signed written consent in compliance with the approved Research Ethics Board (REB) requirements prior to engaging in the consultation exercise. Various engagement techniques were utilized, including reviewing a set of ground rules created by the research team to create an open, inclusive, and welcoming environment for participants during the sessions. Sessions were audio recorded and held via a secure Zoom link. All participants were provided with an honorarium.

The objective of the first session was to determine the differences in importance placed on each LS characteristic between the scoping review results and our consultation exercise group. The characteristics were reviewed briefly without being ranked based on the scoping review. Subsequently, participants engaged in both small and large group discussions to collectively establish consensus on categorizing these characteristics into three groups: (1) very important, (2) moderately important, or (3) less important. Consensus was achieved by deliberating on the importance of each characteristic, with the facilitator encouraging, documenting, and confirming what the group determined as the level of importance for each characteristic.

Following categorization, participants were shown how their priorities differed from the scoping review priorities (represented as the characteristics organized into three groupings, rank ordered from most to least frequently suggested).

The objective of the second and third sessions was to obtain participant perspectives on the suggested processes for writing a LS. First, an infographic was presented which our team created based on scoping review findings to present the processes and their associated steps in an understandable manner (Additional file 3: Fig. S3. Infographic). Second, a large group discussion took place on three questions: “What did the processes in the literature get right? What did they get wrong? and What is missing?” The third session was a continuation of the second but included four additional probing questions for each of the writing processes suggested by participants at the end of the second session: “How can we create the conditions for the involvement of PPPs? What is the best way to involve PPPs in pre-work? In what ways should this process be flexible? and What is missing from the current process?” The intent was to achieve consensus on the preferred steps and to establish guiding principles for each step. After the third session, a summary of the writing processes was shared with the participants as a member checking exercise to ensure accuracy of our summary.

After each consultation exercise session, the research team (i.e., researchers and PPPs) attended one-hour analysis meetings to review and discuss the sessions, review the notes taken and confirm specific plans for subsequent sessions. These analysis meetings were crucial in ensuring the inclusion of the research team PPPs in the analysis of the consultation exercise. The consultation exercise results were a combination of the work done at the sessions and the research team's review of the summary of the session results and activities. Lastly, the final set of characteristics, organized by importance, was compared between the scoping review results and the consultation exercise results.

## Results

**Fig. 1:**
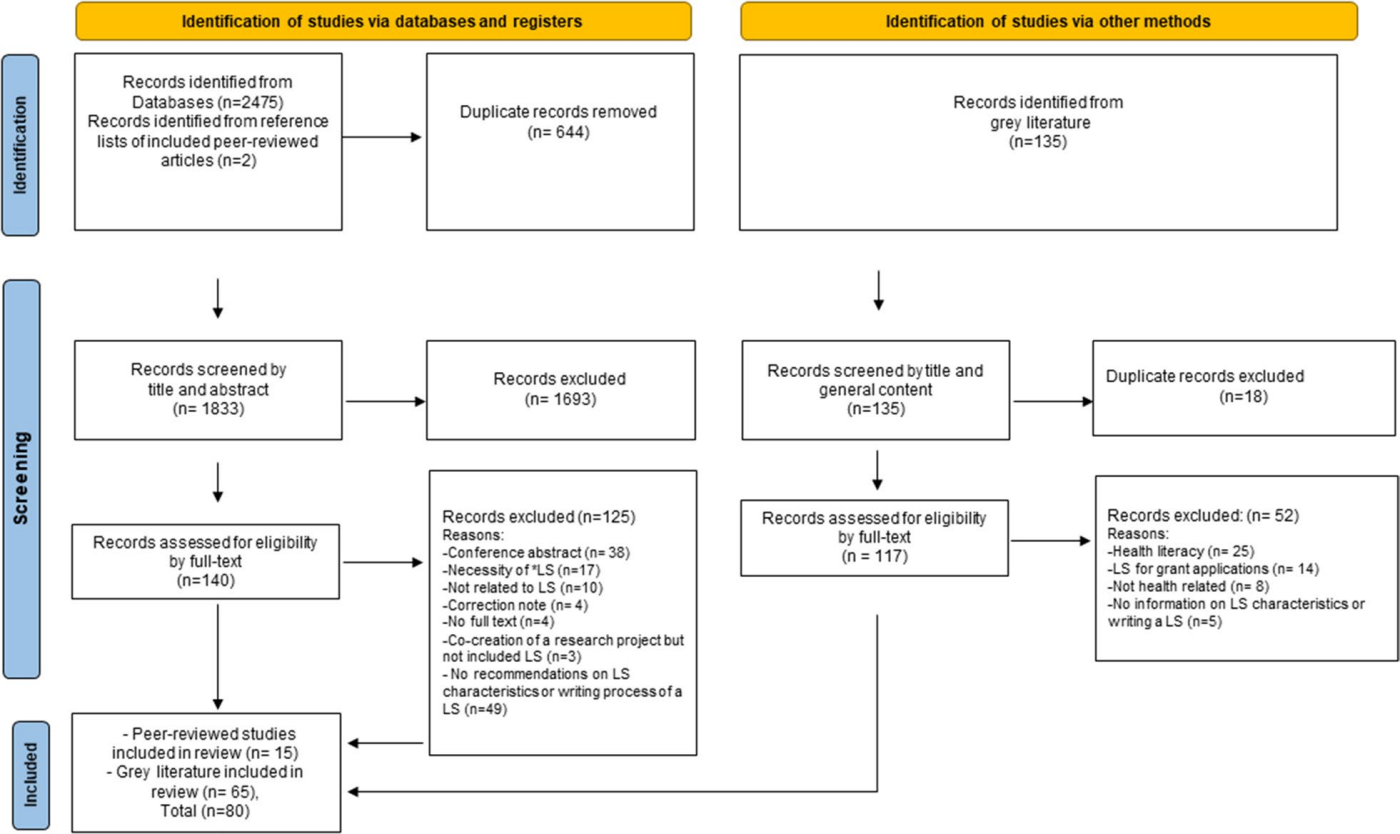
PRISMA Chart. * LS: Lay Summary, (http://www.prisma-statement.org/)

The search process yielded a total of 2,612 sources, including 2,477 peer-reviewed articles and 135 grey literature documents. After removing duplicates, 1950 records were screened based on their title and abstract followed by screening of 257 full text. Of these, 177 documents were excluded with the most common reason for peer-reviewed articles being the lack of recommendations on the LS characteristics and writing processes, and for grey literature, a focus on health literacy. There were 80 resources included in the review: 15 from the peer-reviewed literature and 65 from the grey literature. (Fig. 1 PRISMA chart) (Additional file 4: Table S4, scoping review resources and extracted characteristics/features).

### Description of resources

The majority (n = 65, 80%) of the 80 included resources were from grey literature. The United Kingdom (30%), Canada (24%), and the United States (24%) were the countries with the most resources. The resources were published between 2012 and 2022, with the largest percentage (35%) in 2022. There were 79 (99%) resources recommending LS characteristics and 10 (13%) resources recommending processes for writing a LS. Twenty-two (28%) of resources were specific to a condition, such as Autism or Dermatology, and 30 (38%) were specific to a context, such as clinical trials. There were 76 (95%) resources targeted at researchers and 4 (5%) targeted at other stakeholders including PPPs, policy makers, and funders. PPPs were involved in the creation of 14 (18%) of reviewed resources. See Table 2 for a complete summary of the description of included resources.

**Table 2:** Reviewed resources’ features (n = 80)

Characteristic	n (%)	Characteristic	n (%)
*Source of data*		*Guidance for a specific condition*	
Grey literature	65 (81%)	Yes	22 (28%)
Peer-reviewed	15 (19%)	No	58 (72%)
*Patients and public partners’ involvement in creation of the guidance*		*Guidance for a specific context*	
Yes	14 (18%)	Yes	30 (38%)
No	66 (82%)	No	50 (62%)
*Country of publishing*		*Year of publication*	
United Kingdom	24 (30%)	2022	27 (34%)
Canada	19 (24%)	2021	13 (17%)
United States	19 (24%)	2020	5 (7%)
Norway	4 (5%)	2019	5 (7%)
India	2 (2%)	2018	6 (7%)
Australia	1 (1%)	2017	8 (10%)
Belgium	2(2%)	2016	2 (2%)
Germany	1 (1%)	2015	5(6%)
Iran	1 (1%)	2014	6 (7%)
Japan	1 (1%)	2013	2 (2%)
Mixed	6 (7%)	2012	1 (1%)
Objective of guidance*		*Guidance target audience(s)*	
LS characteristics	79 (99%)	(A) Researchers	76 (95%)
Specific writing process	10 (13%)	(B) Others (patient and public partners,	4 (5%)
Both	9 (11%)	policymakers, funders, unspecified)	

*Some of the resources reported information on both writing lay summary and development process

### Suggested LS characteristics

Thirty-eight LS characteristics were extracted (Table 3). The most often suggested characteristics were avoiding jargon, technical, medical, or scientific language (78%), avoiding complex and long sentences (60%), and using active rather than passive voice (48%). The remaining characteristics were suggested between 30 and 1% of the documents. All characteristics were suggested at least once.

**Table 3:** Frequency of 38 suggested lay summary characteristics ordered by percentage (n = 79)

#	EU-CTR and non-EU-CTR principles	Characteristics	n (%)
1	Health literacy	Avoiding jargon, technical, medical, or scientific language	63 (78%)
2	Health literacy	Removing unnecessary or complex words and/or avoid long sentences	48 (60%)
3	Health literacy	Using active, rather than passive, voice	38 (48%)
4	Health literacy	Using visuals (e.g., simple graphs, tables) to convey messages when helpful	37 (46%)
5	Health literacy	Being consistent in the use of terms/words throughout the document, and define them	35 (44%)
6	Readability	Using a language-specific reading test	24 (30%)
7	Health literacy	Including links to additional information and resources for online summaries and background,	17 (21%)
8	Additional non-EU characteristic	Mentioning funders’/ sponsors’ name(s) in the lay summary	16 (20%)
9	Health literacy	Using bullet points instead of paragraphs	15 (19%)
10	Numeracy	For statistics, presenting absolute numbers but also consider conveying numerical information in other ways such as a percentage, rather than relative risks, odds ratios, etc	12 (15%)
11	Additional non-EU characteristic	Making sure that the lay summary is available relatively soon after the study	12 (15%)
12	Language	Ensuring the summary remain factual and objective	12 (15%)
13	Health literacy	Providing Adequate “white space” (1 or 2 lines)	11(14%)
14	Readability	Providing link(s) to the original study	11 (14%)
15	Readability	Avoiding any promotional language and promotion tone	10 (13%)
16	Additional non-EU characteristic	Avoiding oversimplifying	10 (13%)
17	Visual	Presenting visuals in a simple message with a clear labels and captions and simple textual explanation	10 (13%)
18	Health literacy	Using a glossary in a lay summary	9 (11%)
19	Numeracy	Using words not numbers in results	9 (11%)
20	Additional non-EU characteristic	Mentioning search date/timescale	9 (11%)
21	Health literacy	Presentation of the “big picture” before the detail	7 (9%)
22	Additional non-EU characteristic	Focusing on person not the disability	7 (9%)
23	Language	The summary needs to be provided according to the specific local language of stakeholders and/or the country where the study took place	6 (8%)
24	Language	Including an English version if the trial was published in a non-English language	5 (6%)
25	Language	Translated summaries should also be considered the cultural validity of the medical or technical terminology used	5 (6%)
26	Additional non-EU characteristic	Using sentences in a positive form,	5 (6%)
27	Health literacy	Using the most readable color combination: black text on a white background (Keep in mind how documents will look when online or printed),	4 (5%)
28	Health literacy	Limiting the use of unnecessary imagery that does not enhance understanding,	4 (5%)
29	Health literacy	Avoiding text in ALL CAPS and underlining	4 (5%)
30	Visual	Considering the scales, you are using in any graph and whether the axes need to start at zero to avoid confusion	4 (5%)
31	Visual	Considering creative solutions to ensure understanding could include cartoons and illustrations	4 (5%)
32	Additional non-EU characteristic	Considering LS be indexed in PubMed	4 (5%)
33	Additional non-EU characteristic	Spelling out abbreviations	4 (5%)
34	Visual	Avoiding overly complex images, such as graphs showing several relationships, since they can be easily misinterpreted (e.g., misleading axes labels)	3 (4%)
35	Numeracy	Using whole numbers rather than decimals to the extent. This is possible without increasing confusion should the lay summary be cross-referenced with the scientific summary	3 (4%)
36	Health literacy	Using 12-point font, or ensuring the font size is large enough to read	1 (1%)
37	Visual	Ensuring visuals or graphics are clear enough if printed in black and white	1 (1%)
38	Additional non-EU characteristic	Using inclusive language (do not use she or he)	1 (1%)

### Readability tool, reading level, and word count

A total of 24 resources (30%) recommended the use of readability tools. The most common were Flesch-Kincaid (66%), Simple Measure of Gobbledygook (29%), Readable.io (16.6%) (Table 4).

**Table 4:** Readability tool, n = 24/80

Tool(s)	(n, %)
Flesch-Kincaid	16 (66%)
*SMOG	7 (30%)
Readable.io	4 (17%)
Hemingway	3 (12%)
Gunning Fog	2 (8.5%)
**Others (Mix of readability tools and online readability tools resources)	Readablepro Text readability consensus calculator: http://www.readabilityformulas.com/free-readability-formulatests.php, e.readbility, Readabilityformulas.com/free-readability-formulatests.php, Clear communication index user guide, Fry readability test 3, Lexile framework for reading, PerfectIt™ for microsoft word http://www.readability-score.com/

*SMOG Simple measure of gobbledygook

**Some resources suggested more than one tools

A total of 24 resources (30%) identified reading level as a key characteristic of a LS. The most recommended level was grades 9–12 (high school or ages 14–18) at 21% (Table 5).

**Table 5:** Reading level, n = 24/80

Level(s)	Frequency n (%)
Grade 9–12 (high school, or age 14–18)	5 (21%)
*Undefined	5 (21%) (i.e., ‘grandparent’ level, Indian middle school, Japanese junior high, 8.1 or 6.8–8.5, low to average levels of health literacy)
Grade 6–8	3 (12.5%)
Grade 8	3 (12%)
Grade 8 or lower	2 (8.5%)
Undergraduate level	2 (8.5%)
Grade 8–10	1 (4%)
Grade 12 +	1 (4%)
Grade 6	1 (4%)
Age 11 or older	1 (4%)
Grade 7	1 (4%)

*One of the sources outlined 2 options

A total of 35 (48%) resources provided a specific numerical value for the length of a LS. Fifteen resources (43%) suggested a range of 150–250 words and about fourteen resources (40%) suggested a word limit between 250 and 500 words (Table 6).

**Table 6:** Lay summary number of words, n = 35/80

Range of word numbers	n (%)
150–250	15 (43%)
250–500	14 (40%)
300/500–700/800	5 (14%)
100–1000	1 (3%)

### Content

Fifty-three (66%) resources contained recommendations on the content of a LS (Table 7). The most common included the "What", the main findings of the study, (n = 42, 79%), the "Why", the importance of study, (n = 30, 57%), and the "How", methods, (n = 27, 51%). Six (11%) of these resources indicated that LSs should include the degree of PPPs involvement in the research.

**Table 7:** Content characteristics, n = 53/80

Characteristics	n (%)
What (findings/results)	42 (79%)
Why (importance of study)	30 (57%)
How (methods)	27 (51%)
Implications/relevance	22 (42%)
Who (participants)	15 (28%)
Where	14 (26%)
When (timeline)	12 (23%)
Research questions	7 (13%)
Patient and public partners’ involvement	6 (11%)
Issues/problems the research addresses	6 (11%)
Objectives	6 (11%)

### Processes to write a LS

Ten resources (13%) proposed steps for processes to write a LS. The range of suggested steps in the processes was between 4 and 6. Our summary of the processes yielded six steps: pre-work, preparing audience, writing, reviewing finalizing, and knowledge dissemination. Each step included a range of sub-components (Table 8).

**Table 8:** Lay summary creating processes based on scoping review findings (n = 10/80)

Main steps	Components and their frequencies (n)
Pre-work	Confirm rationale (n = 3)
	Plan needed human and budget resources (n = 3)
	Plan what LS should look like (format, characteristics) (n = 3)
	Plan where to publish, author group, dissemination plan (n = 2)
	Plan timelines, action items (n = 1)
Audience preparation	Determine audience (n = 6)
	Enlist stakeholders (n = 3)
	Create an advisory board (n = 1)
Writing	Researcher writes LS (n = 6)
	Researchers engages PPP in writing a LS (n = 2)
	Researchers + PPP writes LS in a workshop (n = 1)
	Practice explaining the research (n = 1)
	Read the publication (n = 1)
Reviewing	Non-specialist or PPP (n = 3)
	In house (n = 2)
	Focus group(s) with PPP (n = 2)
	Focus group with advisory board (n = 1)
	Read aloud (n = 1)
	PPP advocacy group (n = 1)
	Cognitive testing (broad group including PPP) (n = 1)
Finalizing	Finalize with researchers (n = 4)
	Use production team to finalize the LS (n = 3)
	Readability testing (n = 2)
	Characteristic checklist (n = 1)
	Create translation if needed (n = 1)
	Finalize with audience and researchers (n = 1)
Knowledge dissemination	Disseminate (e.g., audio or written) (n = 4)

### Consultation exercise

Twelve participants (eight PPPs, four researchers) responded to the recruitment efforts, and all met the inclusion criteria. Session one was attended by seven PPPs and four researchers, session two by seven PPPs and four researchers, and session three by six PPPs and three researchers. Nine out of 12 participants made it to all sessions. Not all participants were able to attend due to scheduling conflicts.

### Session 1: LS characteristics

Our participant group categorized 16 characteristics as the most important, 10 as moderately important, and 12 as less important. Table 9 summarizes detailed information on how participants categorized the 38 LS characteristics compared to the literature in the scoping review. Participants agreed with literature regarding the importance of “avoiding jargon,” “using long and complex sentences,” “defining terms,” “using visuals,” “making LSs available after study,” and “being factual and objective.”

**Table 9:** Comparing the importance of the EU characteristics between literature results and participants’ opinions

EU characteristics (Top 3rd)	Very Important	Moderately Important	Less Important	EU characteristics (Middle 3rd)	Very Important	Moderately Important	Less Important	EU characteristics (Bottom 3rd)	Very Important	Moderately Important	Less Important
No jargon	P, L			Link to study paper	P	L		Black and white			P, L
No long/complex sentence	P, L			Avoid promotional language		P(d), L		No unnecessary images		P	L
Active Voice	P, L			Don’t oversimplify	P	L		No CAPS or underline			P, L
Define terms	P, L			Glossary	P	L		Graphs having a ‘0’		P	L
Use visuals	P, L			Words not numbers in results		L	P	Cartoons and Illustrations			P, L
Use reading test	L		P	Timeline		L	P	Index in PubMed			P, L
Links to additional info	L		P	Big picture first	P(d)	L		Spell out abbreviations	P		L
State funder	P			Person, not disability	P(d)	L		Avoid complex images	P		L
Bullets	L	P(d)		Local language	P	L		No decimals			P, L
Use absolute numbers	L		P	English version		P, L		12-point font			P, L
Available soon	P, L			Culturally valid	P	L		Visuals clear in print			P, L
Factual/objective	P, L			Label/describe visuals	P	L		Inclusive language	P		L
White space	L	P(d)		Positive wording		P, L					

*P* Participants perspectives, *L* Literature, *d* Depends on

However, some characteristics that were considered less important in the literature were deemed important by our participants including “link to the original study paper”, “big picture first”, “mentioning funders”, “having glossary”, “LS in a local language”, “culturally valid LS”, “labeling visuals”, “person not a disability”, “positive wording”’, “spell out abbreviations”, “avoiding complex image” and “inclusive language”. Participants also expressed a different perspective on the use of reading tests. The literature suggested the use of these tests 30% of the time yet our consultation exercise participants rated them in the lowest category (less important). The group indicated that reading tests are an inadequate way of measuring readability and understanding. Instead, they suggested that it is more critical to ensure that the LS can be understood by the intended audience. Therefore, having someone from the target audience review the LS for comprehension would be a better approach than using readability tests.

Despite being able to reach consensus on the categorization by importance, participants expressed that these categories were overly simplistic and often indicated caveats to the categorization (represented as “d” for “it depends” in Table 9). For example, the characteristic “person, not disability" may not always apply to communities where identity takes precedence. The group indicated that while being “non-promotional” is important, there may be times when a message for the public good should be emphasized. The characteristics related to font or printing quality were less important if the LS is accessed online. Even the highly rated (by both the scoping review and the consultation exercise) "avoid jargon" was deemed too restrictive because participants indicated that jargon is unavoidable, and that learning jargon can actually help in self-advocacy efforts. The characteristic "avoid complex images" was also considered too simplistic and could be better phrased as "be cautious with images and add alternative text when including them."

### Sessions 2 and 3: LS writing processes

Participants emphasized the LS writing process more important than the list of characteristics. One of the most important issues discussed was the need for all studies to include a LS. As well, to create an environment that validates and legitimizes the role of PPPs and to offer them the opportunity to be in a desired role in writing a LS. They expressed that a power balance is needed between researchers and PPPs to achieve a successful outcome and their token participation was unacceptable.

The participants agreed with the literature's findings on the pre-work required before writing a LS, particularly for understanding the target audience. However, they emphasized that PPPs should be involved in every step of the LS writing processes, such as being included in the dissemination of a LS and that researchers should never write a LS alone. Further, they believed that in-house reviewing of the LS would never be sufficient and that having reviewers naïve to the project was critical. Additionally, they suggested that a characteristic checklist would generally be unhelpful. Instead, they proposed a list of guiding questions for a LS, such as "What did you do? What did you find? Why does it matter?".

In the third session, participants reached consensus on a revised set of steps for writing a LS. The original six steps outlined in the literature (pre-work, audience preparation, writing, reviewing, finalizing, disseminating) were modified into six different steps (two steps were combined and one step was added): Preparing (includes pre-work and preparing audience), Writing, Reviewing, Finalizing, Disseminating and Evaluating. The last step of evaluation was added and applies to both the LS and its writing processes. Table 10 shows a comprehensive summary of the six recommended steps for writing a LS and their corresponding principles.

**Table 10:** Consultation exercise sessions 2 and 3—key principles and writing processes

Steps	Key principles from consultation exercise participants’ perspectives
1. Preparing	PPP should be involved in every step of the process including preparation and preferably engaged in a leadership or co-leadership role from the onset of the development of the *LS
	Ideally, a team should have at least two **PPP and work to create a safe, comfortable partnership for all team members
	PPPs should be engaged in a discussion about the kinds of LS writing skills they would like to contribute and acquire
	It is useful to consider the following two groups: the writing team and the target audience. The first task is to compose the writing team that includes PPPs, then as a team, determine the primary audience the team is writing for
	Researchers working with the PPP on the LS need to consider, and plan for, how they will support PPP members to develop the LS writing skills they are interested in acquiring
	Determine the purpose of the LS and the audience for the LS at the same time—these two considerations go together
2. Writing	The writing team should continue to follow through on their plan outlined during in step 1 while being open to any accommodations that may need to be made
	Confirm a format for the LS. Having a sample template is very helpful for PPPs to draft LSs
	Offer the PPPs a chance to write the LS
	Guiding questions can be helpful for writing such as “What did you do, what did you find, why does this matter?”
	Ask PPPs to review the study and ask them “What do you think is the most important information to communicate?”
3. Reviewing	Every PPP on the team should be given the opportunity to review the drafts of the LS
	Read the LS aloud
	Conduct user testing by showing the LS to 3–5 people who are representative of the main audience, but are not members of the LS team, and request detailed feedback from them. This feedback is essential as the writing team may be too close to the material to evaluate its readability and comprehensiveness
	Focus groups are not necessarily needed for reviewing
4. Finalizing	This is an important step, and it is different than reviewing
	Conduct a final review to ensure there is no misrepresentation of the study
	Finalization may require additional rounds of user testing (e.g., to ensure proper translations)
	A production team can be useful for design and incorporating images and captions, but should prioritize accessibility (e.g., screen reader friendly, all visuals have alt text)
5. Disseminating	Consider what format(s), for example hard-copy, digital or audio, the final LS will be disseminated in. Intended audience(s) might dictate dissemination
	Need a specific dissemination team to facilitate planning and ensure accessibility (e.g., creating a social media campaign)
6. Evaluating	This step should be taken once the LS has been disseminated
	Evaluate the LS: Did it ‘work’ as it was originally intended? Consider whether any metrics may be collected to support the LS’ evaluation (e.g., numbers of downloads, accesses on a website, etc.)?
	Evaluate the process used by the writing team to create the LS: Did the writing process work well? Could it be improved for next time? What did the writing team members take away from their experiences working together on the LS? Would they be open to working together again? Consider having PPP and researchers who have experience collaborating on the development of a LS be future mentors for a next group who will work on a similar task

*LS Lay summary

**PPP Patient and public partner

## Discussion

The objectives of this scoping review with a consultation exercise were to delineate the features of the available LS resources, summarize the recommended LS characteristics and content, synthesize the recommended LS writing processes, and gather PPPs and researchers' perspectives on the review findings. To our knowledge, this review was the first to synthesize LS characteristics and writing processes. The project was also novel as it was initiated by a PPP and co-led by a PPP and a researcher. Using an iKT approach facilitated the involvement of our PPPs across all steps of the project (table GRIPP 2) [30]. Employing the consultation exercise to contextualize the review findings furthered the inclusion of diverse perspectives of knowledge users and facilitated our co-creation by actively engaging PPPs and research participants in rank ordering important LS characteristics and elaborating LS wring processes [13].

Our review showed that the majority of resources focused on LS characteristics as opposed to the writing process, and more than half of the resources were published in the last two years. The most frequently suggested LS content was to include study findings. Very few resources were targeted at PPPs or included PPPs in their development. The prioritization of LS characteristics by consultation exercise participants differed from that of the literature in terms of their importance, with many participants finding certain characteristics over simplistic. The consultation exercise participants emphasized that a one-size-fits-all checklist of characteristics may not be helpful, as LS characteristics often depend on contextual factors and the needs of the target audience. Although few studies specified LS writing processes, our consultation exercise resulted in the proposal of a six-step process for writing a LS.

### LS features

The higher rate of producing LS resources between 2020 and 2022 in North America and Europe might indicate a growing interest in the topic [15]. Further, the higher percentage of resources found in grey literature as compared to peer-reviewed articles underscores the valuable insights that might be more accessible to PPPs [31], which is important because PPPs need access to LS guidance to engage in LS creation [14]. Conversely, while focusing on creating more peer-reviewed studies may potentially slow down evidence creation, the scientific rigor and scrutiny involved in peer-reviewed evidence ensures a higher level of credibility and validity [32, 33]. The lack of peer-reviewed literature could also reflect researchers’ challenges in focusing on LSs as they are trained and accustomed to writing for subject specialists or academics, rather than the public or non-specialist audience [34].

Illustrating the importance of stakeholder perspectives, our consultation exercise participants indicated that having resources that elaborate the LS writing process was more valuable than a list of recommended LS characteristics, contrasting with the review results, which indicated the opposite in terms of available resources (i.e., recommendations on LS characteristics was 99% and writing processes was 13%). The literature’s lack of emphasis on the writing process could potentially hinder the overall quality and impact of LS production, particularly if researchers only access peer-reviewed resources for LS guidance.

Our study was able to describe the extent of PPPs’ involvement in LS guidance (18% of the available resources were produced in part with PPPs and 5% were specifically intended for PPPs). Previous reviews on LSs did not report PPP involvement [15, 17] making comparisons to other literature difficult, however, our team PPPs indicated this was a common omission. Considering the significant emphasis our consultation exercise participants placed on PPP involvement, this area requires further attention, particularly related to PPP roles in creating LS resources and their participation in the writing processes. Understanding the potential benefits, challenges, and strategies associated with PPPs collaboration in developing LS guidance and crafting a LS is imperative to develop more effective and impactful partnerships that facilitate PPPs’ engagement, while promoting equitable access to LS resources [2, 14].

### Recommended LS characteristics

Our findings revealed that only two characteristics were recommended by more than 50% of included resources (i.e., “avoid jargon” and “avoid complex sentences”), implying limited consensus on other optimal LS characteristics. Results on these two characteristics and findings on “content” (i.e., using what, where, who, when, or how questions), and “word count range” (i.e., a wide varied range of words from 150 to 1000) were congruent with previous review studies on LS resources [15, 17, 35].

By utilizing the EU-CTR to extract data on LS characteristics, we were able to create some structure around the many LS characteristics that exist and took the opportunity to expand the list of potential (and possibly important) characteristics. Further efforts to build on the EU-CTR framework could lead to an even more robust approach to identifying and structuring characteristics.

Contrary to the literature findings, in our study, consultation exercise participants expressed reservations regarding the sole reliance on reading level and readability tools as a comprehensive strategy to ensure the appropriateness of a LS for diverse audiences. By directly engaging the intended audience in reviewing LS materials, communicators can gain valuable insights into the clarity, comprehensibility, and relevance of the content. This approach acknowledges the inherent variability in audience backgrounds, prior knowledge, and language abilities, and allows for tailored adjustments to optimize LS comprehension and foster PPP engagement [2, 14].

### Processes to write a LS

The contributions of the consultation exercise participants in contextualizing our review findings were instrumental to our review and shaped much of what was found. One notable suggestion from the consultation exercise participants was to include a LS for all studies. Making a LS an essential component of all studies aligns with the growing recognition of the importance of health evidence communication with public audiences which encourages researchers to actively consider the needs of various audiences throughout the research process and to effectively communicate their findings in a manner that is accessible and comprehensible to a wider range of individuals [13, 35–38].

Consultation exercise participants insights led to a more comprehensive framework for LS writing processes including the integration of “pre-work" and "preparing audience" steps within the LS writing process. This recognizes the importance of upfront planning, assembling the LS writing team, and understanding the target audience before embarking on writing. Furthermore, the consultation exercise participants emphasized the inclusion of an "evaluation" step within the LS writing process. This addition acknowledges the significance of assessing the LS as an output, evaluating its creation processes and the functions it serves. This evaluation allows for necessary adjustments, contributing to the enhancement of future LSs. Previous reviews have not focused on LS processes [15, 17] and only 13% of our included sources contained recommendations on the process of writing a LS with focusing on different areas. For instance, Dormer et al. [39] suggested process steps such as preparation, writing and reviewing, and dissemination. They suggested researchers should be the main writer of a LS with PPPs as reviewers [39]. Maurer et al.’s [40] main focus was on writing and dissemination steps rather than any preparation and evaluation. Our consultation exercise participants indicated that writing processes may be one of the most important aspects and their inclusion of an evaluation step to the process is insightful. Additional efforts to advance our understanding of optimal processes and evaluation of processes to write a LS is critical.

The consultation exercise participants stressed the need for a collaborative approach, where researchers and PPPs work together as equal partners in the LS writing process. This collaborative mindset fosters an environment of mutual respect, shared decision-making, and open dialogue [13, 36]. They highlighted the significance of recognizing and addressing the common power imbalances that may exist within team dynamics [41, 42] to ensure that all team members have an equal voice and are actively involved in shaping the content and direction of the LS creation.

## Limitations and future directions

Despite implementing a comprehensive search strategy, adhering to the PRESS search strategy criteria [25], and adopting the NIHR definition for “lay summary,” the lack of consensus regarding the terminology used to denote a "lay summary” may have resulted in missed resources, particularly for the grey literature. Using the EU-CTR framework for LS characteristics helped structure our extraction and while we did add additional variables deemed important during extraction, there could still be other important characteristics of LSs that were not included. The contribution of consultation exercise participants enhanced the relevance and applicability of the study's findings and conclusions by contextualizing the identified characteristics and writing processes with the needs and expectations of PPPs, however, it was only with a small group of participants. A larger group or a different group may have come to different conclusions. In order to focus on LSs more broadly, we did not extract information from the sources that were specific to condition or context. It is possible that this more specific information could enhance what is known about LS characteristics, and while we can assume that our broad results apply to specific contexts and conditions, we are not certain of the degree of this application. The science of lay summaries appears to be in its infancy. Future work in multiple areas is essential to shed light on how best to engage PPPs and any knowledge user or lay person in the creation and evaluation of LSs, best practices for LS characteristics and for the process of writing a LS would be useful steps towards ensuring that anyone can access scientific evidence.

## Conclusions

This scoping review with a consultation exercise provided invaluable information on available resources regarding LS characteristics and writing processes. This study recognizes the imperative of involving PPPs in the process of writing a LS to advance the effective communication of healthcare evidence. Additionally, one output of this study (i.e., key principles to engage PPPs in the LS writing processes) is a contribution to enhancing the principles of LS writing.

### Supplementary Information

**Additional file 1: Table S1.** Data bases search strategy.

**Additional file 2: Fig. S2.** Roadmap of conducting a scoping review with consultation exercise.

**Additional file 3: Fig. S3.** Infographic prepared to present processes for writing a LS to CE Participants.

**Additional file 4: Table S4.** List of resources included in this scoping review.

## Data Availability

All data are provided in tables and supplemental files.

## Abbreviations

LSs: Lay summaries
PPPs: Patient and Public Partners
PPs: Patient partners
EU-CTR: European Clinical Trial Regulation
SPOR-EA: Strategy for Patient-Oriented Research-Evidence Alliance
