# Supplementary material for: Recommended characteristics and processes for writing lay summaries of healthcare evidence: a co-created scoping review and consultation exercise
Source: Res Involv Engagem. 2023 Dec 20;9:121. doi: 10.1186/s40900-023-00531-5 (PMC10734197; doi:10.1186/s40900-023-00531-5)
Supplement: Supplementary file 1 — Additional file 1: Table S1. Data bases search strategy. [file 40900_2023_531_MOESM1_ESM.docx]

S1 Table. Data bases search strategy

**Ovid MEDLINE**: Epub Ahead of Print, In-Process & Other Non-Indexed Citations, Ovid MEDLINE® Daily and Ovid MEDLINE® <1946-Present>, Embase Classic+Embase <1947 to date>, APA PsycInfo <1806 to present> Journals@Ovid Full Text

| 1 | "plain language summar*".ti,kf. |
| --- | --- |
| 2 | "plain English summar*".ti,kf. |
| 3 | "lay summar*".ti,kf. |
| 4 | "lay English summar*".ti,kf. |
| 5 | "results summar*".ti,kf. |
| 6 | "non technical description*".ti,kf. |
| 7 | "non-technical description*".ti,kf. |
| 8 | "nontechnical description*".ti,kf. |
| 9 | "lay language summar*".ti,kf. |
| 10 | "plain-language abstract*".ti,kf. |
| 11 | "lay abstract*".ti,kf. |
| 12 | ((Writ* or develop* or creat*) adj2 lay summar*).ab. |
| 13 | ((Writ* or develop* or creat*) adj2 plain-language summar*).ab. |
| 14 | ((Writ* or develop* or creat*) adj2 plain English summar*).ab. |
| 15 | ((Writ* or develop* or creat*) adj2 lay English summar*).ab. |
| 16 | ((Writ* or develop* or creat*) adj2 non technical description*).ab. |
| 17 | ((Writ* or develop* or creat*) adj2 nontechnical description*).ab. |
| 18 | ((Writ* or develop* or creat*) adj2 lay language summar*).ab. |
| 19 | ((Writ* or develop* or creat*) adj2 plain-language abstract*).ab. |
| 20 | ((Writ* or develop* or creat*) adj2 lay abstract*).ab. |
| 21 | 1 or 2 or 3 or 5 or 6 or 7 or 8 or 9 or 11 or 12 or 13 or 14 or 20 |
| 22 | limit 21 to English language |
| 23 | limit 22 to human |
| 24 | limit 23 to humans |

**Cochrane**

| #1 | ("plain language summary"):ti OR ("lay summary"):ti OR ("result summary"):ti OR ("non technical summary"):ti OR ("lay person summary"):ti in Cochrane Reviews, Cochrane Protocols, Trials, Clinical Answers, Editorials, Special Collections (Word variations have been searched) |
| --- | --- |
| #2 | (lay NEAR/3 (summary OR summaries)):ti,kw |
| #3 | (plain language NEAR/3 (summary OR summaries)):ti,kw |
| #4 | (plain English NEAR/3 (summary OR summaries)):ti,kw |
| #5 | (plain-language NEAR/3 (summary OR summaries)):ti,kw |
| #6 | (lay language NEAR/3 (summary OR summaries)):ti,kw |
| #7 | (lay English NEAR/3 (summary OR summaries)):ti,kw |
| #8 | (lay person NEAR/3 (summary OR summaries)):ti,kw |
| #9 | (result NEAR/3 (summary OR summaries)):ti,kw |
| #10 | (non-technical NEAR/3 (summary OR summaries)):ti,kw |
| #11 | (nontechnical NEAR/3 (summary OR summaries)):ti,kw |
| #12 | (non technical NEAR/3 (summary OR summaries)):ti,kw |
| #13 | (non-technical NEAR/3 (description OR descriptions)):ti,kw |
| #14 | (nontechnical NEAR/3 (description OR descriptions)):ti,kw |
| #15 | (non technical NEAR/3 (description OR descriptions)):ti,kw |
| #16 | (nontechnical NEAR/3 (description OR descriptions)):ti,kw |
| #17 | #1 OR #2 OR #3 OR #5 OR #6 OR #9 OR #10 OR #13 |
| #18 | (plain language NEAR/3 (abstract OR abstracts)):ti,kw |
| #19 | (lay NEAR/3 (abstract OR abstracts)):ti,kw |
| #20 | #17 OR #18 in Cochrane Reviews, Cochrane Protocols, Trials, Clinical Answers, Editorials, Special Collections (Word variations have been searched) |
| #21 | (Writ* or develop* or creat*):ti,kw AND #20 |
| #22 | #20 OR #21 |

**CINHAL**

| S1 | TI"plain-language summar*" OR ( TI "plain English summar*" OR TI "lay summar*" OR TI "lay English summar*" OR TI "lay person summar*" OR TI "results summar*" OR TI "non-technical summar*" OR TI "non technical description*" OR TI "non technical summar*" OR TI "non-technical description*" OR TI "lay language summar*" ) ) OR ( ((AB "plain-language summar*" OR AB "plain English summar*" OR AB "lay summary*" OR AB "lay English summary*" OR AB "lay person summary*" OR AB "results summar*" OR AB "non-technical summar*" OR AB "non technical description*" OR AB "non technical summary*" OR AB "non-technical description*" OR AB "lay language summar*") ) |
| --- | --- |
| S2 | TI ( (Writ* or develop* or creat*) ) OR AB ( (Writ* or develop* or creat*) ) |
| S3 | #S1 and #S2 |

**PubMed**

"plain language summary*"[Title/Abstract] OR "plain english summary*"[Title/Abstract] OR "lay summary*"[Title/Abstract] OR "results summary*"[Title/Abstract] OR "non-technical summary*"[Title/Abstract] OR "nontechnical summary*"[Title/Abstract] OR "non-technical description*"[Title/Abstract] OR (("lay summary*"[Title] AND ("writ*"[Title] OR "develop*"[Title] OR "creat*"[Title)) OR ("plain language summary*"[Title] AND ("writ*"[Title] OR "develop*"[Title] OR "creat*"[Title])) OR ("non technical summary*"[Title] AND ("writ*"[Title] OR "develop*"[Title] OR "creat*"[Title])) OR ("non-technical summary*"[Title] AND ("writ*"[Title] OR "develop*"[Title] OR "creat*"[Title] OR ("nontechnical summary*"[Title] AND ("writ*"[Title] OR "develop*"[Title] OR "creat*"[Title]))))) Filters: Clinical Trial, Meta-Analysis, Randomized Controlled Trial, Review, Systematic Review, Humans, English

**Scopus**

TITLE ( ( "plain language summar*" )  OR  ( "plain English summar*" )  OR  ( "lay summar*" )  OR  ( "lay English summar*" )  OR  ( "lay person summar*" )  OR  ( "results summar*" )  OR  ( "non-technical summar*" )  OR  ( "non technical description*" )  OR  ( "non technical summar*" )  OR  ( "non-technical description*" )  OR  ( "lay language summar*" )  OR  ( "plain-English summary*" )  OR  ( "lay abstract*" )  OR  ( "plain language abstract*" ) )  OR  TITLE-ABS ( ( writ*  OR  develop*  OR  creat* )  W/2  "plain language summar*" )  OR  TITLE-ABS ( ( writ*  OR  develop*  OR  creat* )  W/2  "plain English summar*" )  OR  TITLE-ABS ( ( writ*  OR  develop*  OR  creat* )  W/2  "lay summar*" )  OR  TITLE-ABS ( ( writ*  OR  develop*  OR  creat* )  W/2  "lay summar*" )  OR  TITLE-ABS ( ( writ*  OR  develop*  OR  creat* )  W/2  "lay person summar*" )  OR  TITLE-ABS ( ( writ*  OR  develop*  OR  creat* )  W/2  "results summar*" )  OR  TITLE-ABS ( ( writ*  OR  develop*  OR  creat* )  W/2  "non-technical summar*" )  OR  TITLE-ABS ( ( writ*  OR  develop*  OR  creat* )  W/2  "nontechnical summar*" )  OR  TITLE-ABS ( ( writ*  OR  develop*  OR  creat* )  W/2  "non technical summar*" )  OR  TITLE-ABS ( ( writ*  OR  develop*  OR  creat* )  W/2  "non technical description*" )  AND  ( LIMIT-TO ( LANGUAGE ,  "English" ) )

**ERIC**

[ti(("plain language summary*") OR ("plain English summary*") OR ("lay summary*") OR ("lay English summary*") OR ("lay person summary*") OR ("results summary*") OR ("non-technical summary*") OR ("non technical summary*") OR ("nontechnical summary*") OR ("non technical description*") OR ("non-technical description*") OR ("nontechnical description*") OR ("lay language summary*") OR ("plain-language abstract*") OR ("lay abstract*")) OR ab(( ( writ* OR develop* OR creat* ) and "plain language summar*" ) OR ( ( writ* OR develop* OR creat* ) and "plain English summar*" ) OR ( ( writ* OR develop* OR creat* ) and "lay summar*" ) OR ( ( writ* OR develop* OR creat* ) and "lay summar*" ) OR ( ( writ* OR develop* OR creat* ) and "lay person summar*" ) OR ( ( writ* OR develop* OR creat* ) and "results summar*" ) OR ( ( writ* OR develop* OR creat* ) and "non-technical summar*" ) OR ( ( writ* OR develop* OR creat* ) and "nontechnical summar*" ) OR ( ( writ* OR develop* OR creat* ) and "non technical summar*" ) OR ( ( writ* OR develop* OR creat* ) and "non technical description*" ) )](https://www.proquest.com/recentsearches.recentsearchtabview.recentsearchesgridview.scrolledrecentsearchlist.checkdbssearchlink:rerunsearch/317B1C4F13114E7BPQ/None?site=eric&t:ac=RecentSearches)Limits applied

**PsychInfo (Psychology)**

"plain language summar*" OR "plain English summar*" OR "lay summar*" OR "lay English summar*" OR "lay person summar*" OR "results summar*" OR "non-technical summar*" OR "non technical summar*" OR "nontechnical summar*" OR "non technical description*" OR "non-technical description*" OR "nontechnical description*" OR "lay language summar*" OR "plain-language abstract*" OR "lay abstract*"

OR (writ* OR develop* OR creat*) AND "plain language summar*" OR (writ* OR develop* OR creat*) AND "plain English summar*" OR (writ* OR develop* OR creat*) AND "lay summar*" OR (writ* OR develop* OR creat*) AND "lay summar*" OR (writ* OR develop* OR creat*) AND "lay person summar*"

Limited to: Scholarly Journals

1980-2022, English
