# Supplementary figures and images for: Recommended characteristics and processes for writing lay summaries of healthcare evidence: a co-created scoping review and consultation exercise
Source: Res Involv Engagem. 2023 Dec 20;9:121. doi: 10.1186/s40900-023-00531-5 (PMC10734197; doi:10.1186/s40900-023-00531-5)

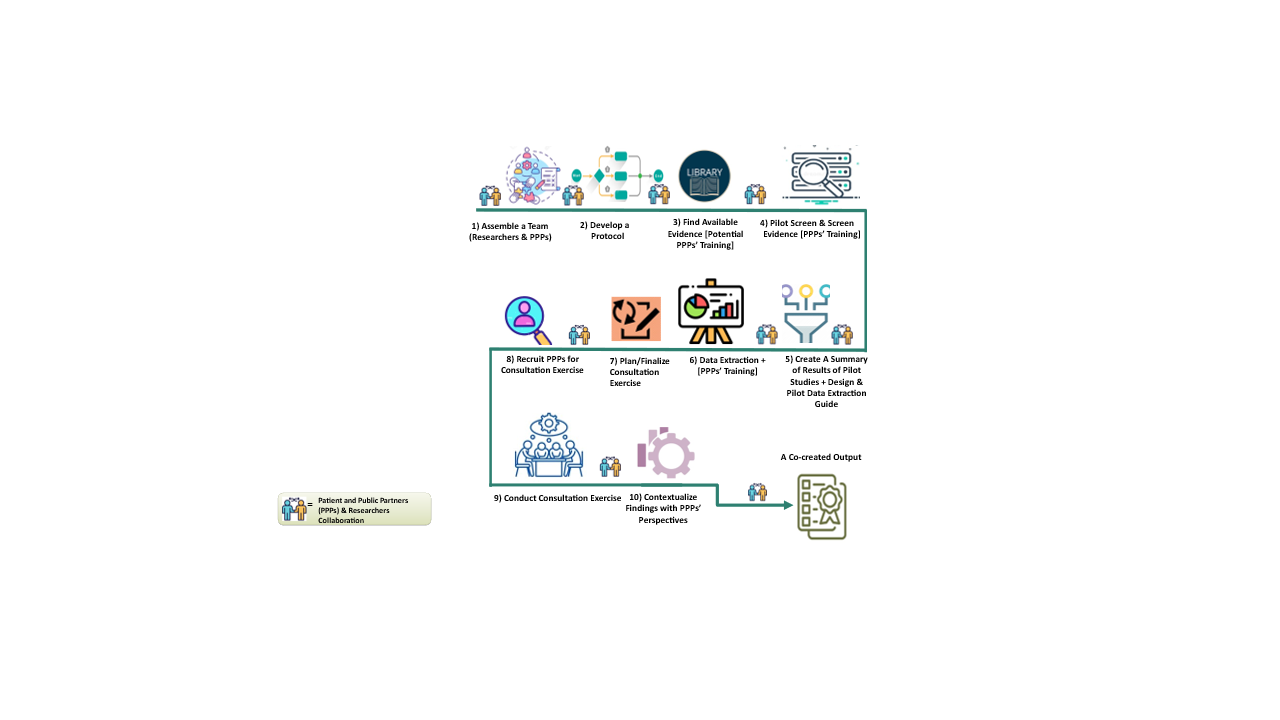

Supplement: Supplementary file 2 — Additional file 2: Fig. S2. Roadmap of conducting a scoping review with consultation exercise. [file 40900_2023_531_MOESM2_ESM.tif]

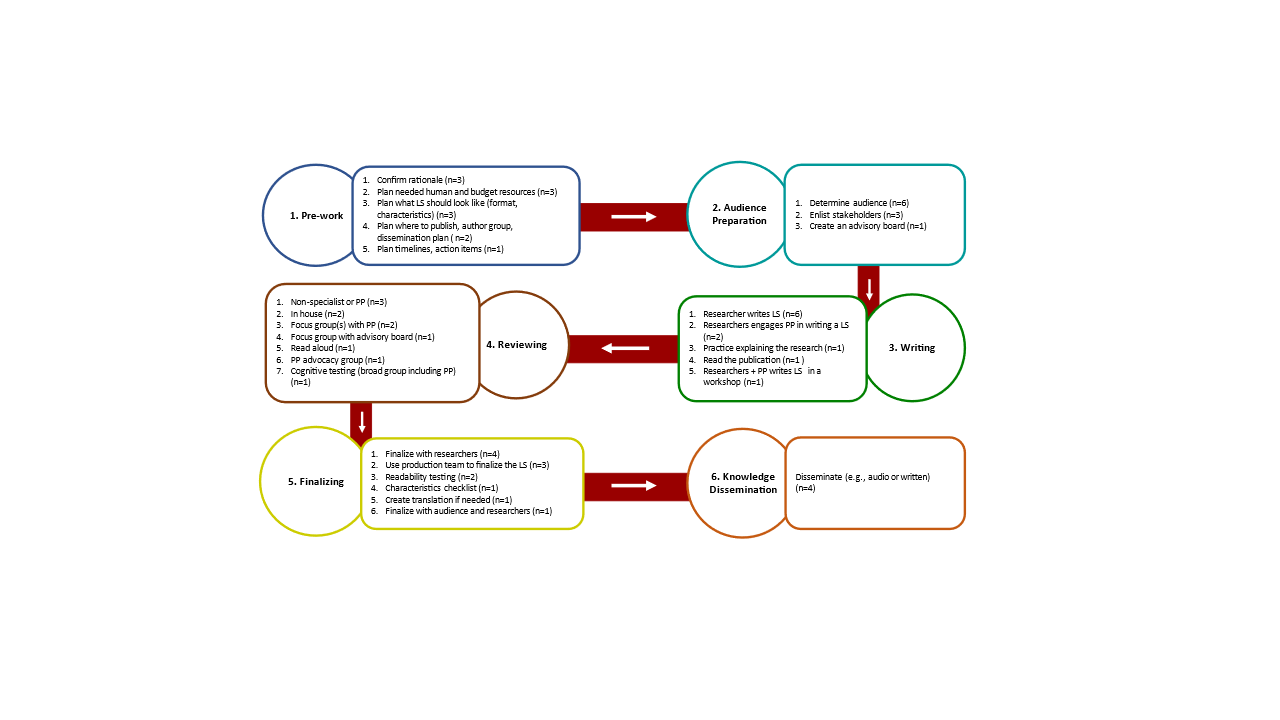

Supplement: Supplementary file 3 — Additional file 3: Fig. S3. Infographic prepared to present processes for writing a LS to CE Participants. [file 40900_2023_531_MOESM3_ESM.tif]
