# Supplementary material for: Recommended characteristics and processes for writing lay summaries of healthcare evidence: a co-created scoping review and consultation exercise
Source: Res Involv Engagem. 2023 Dec 20;9:121. doi: 10.1186/s40900-023-00531-5 (PMC10734197; doi:10.1186/s40900-023-00531-5)
Supplement: Supplementary file 4 — Additional file 4: Table S4. List of resources included in this scoping review. [file 40900_2023_531_MOESM4_ESM.docx]

| **#** | **Type of sources** | **Resources and Publications** | **Year** | **Extracted information** |
| --- | --- | --- | --- | --- |
|  | Grey literature | Advancing Earth and Spacing Science, Summarizing Your Science, <https://www.agu.org/Publish-with-AGU/Publish/Author-Resources/Plain-Language-Summary> | 2022 | EU-CTR characteristics, *LS content, LS number of words, Reading level |
|  | Grey literature | Agriculture and Agri-Food Canada, Agriculture and Agri-Food Canada Policy on Science and Technology Publications-Appendix C: Plain Language Summary guidelines, <https://agriculture.canada.ca/en/agricultural-science-and-innovation/agriculture-and-agri-food-canada-science-integrity-policy/agriculture-and-agri-food-canada-policy-science-and-technology-publications#appendixc> | 2021 | EU-CTR characteristics, LS content, LS number of words |
|  | Grey literature | Alberta SPOR Support-Lay Summaries, Writing Research in Plain English, <https://albertainnovates.ca/app/uploads/2018/02/WritingLaySummaries.pdf> | 2018 | EU-CTR characteristics, LS content, Reading tool, |
|  | Grey literature | American Chemistry Society, Writing for the Lay Public: Engaging  and Educating the General Population, <https://www.acs.org/content/dam/acsorg/events/professional-development/Slides/2020-4-20-writing-rebroadcast.pdf> | 2017 | EU-CTR characteristics, |
|  | Grey literature | American Psychological Association, Guidance for writing a public significance statement, <https://www.apa.org/pubs/journals/resources/translational-messages> | 2018 | EU-CTR characteristics, LS content,, LS number of words |
|  | Grey literature | Arthropod Ecology-McGill University, A guide for writing plain language summaries of research papers, <https://arthropodecology.com/2013/08/01/a-guide-for-writing-plain-language-summaries-of-research-papers/> | 2013 | EU-CTR characteristics, LS content,, LS number of words |
|  | Grey literature | Australian Clinical Trials Alliance, Writing in plain language, <https://involvementtoolkit.clinicaltrialsalliance.org.au/toolkit/undertaking/writing-in-plain-language/> | 2022 | EU-CTR characteristics, LS content, Reading tool, Reading level, |
|  | Grey literature | Autism's Own Journal, Submission Preparation checklist. <https://journals.uvic.ca/index.php/autismsown/about/submissions> | 2022 | EU-CTR characteristics, LS number of words |
|  | Grey literature | Beatrice Hunter Cancer Research Institute, Tips for Writing Plain Language Summaries, <https://bhcri.ca/assets/components/resources/docs/Tips%20for%20lay%20summaries.pdf> | 2022 | EU-CTR characteristics, LS content, Reading tool, Reading level, |
|  | Grey literature | British Ecology Society, Plain Language Summary Guidelines, <https://www.britishecologicalsociety.org/wp-content/uploads/2020/07/Plain-Language-Summary-Guidelines_2020.pdf> | 2022 | EU-CTR characteristics,, LS number of words |
|  | Grey literature | Campbell Collaboration, How to write a plain language summary for a Campbell systematic review, <https://core.ac.uk/download/pdf/52568807.pdf> | 2016 | EU-CTR characteristics, LS content, LS number of words |
|  | Grey literature | Canadian Institute for Military and veteran Research, Writing The LS Basics, <https://cimvhr.ca/forum/resources/WritingTheLaySummaryBasics.pdf> | 2022 | EU-CTR characteristics, LS content, LS number of words |
|  | Grey literature | Canadian dermatology foundation, How to Prepare a Lay Abstract, <https://cdf.ca/en/how-to-prepare-a-lay-abstract/> | 2022 | EU-CTR characteristics, LS content, LS number of words |
|  | Grey literature | Canadian Frailty network, Guidelines for Writing a LS, <https://www.cfn-nce.ca/wp-content/uploads/2017/09/cfn-guidelines-for-lay-summaries.pdf> | 2017 | EU-CTR characteristics, LS content, Reading tool, Reading level, LS number of words |
|  | Grey literature | Canadian Science Publishing, Writing a Plain Language Summary, <https://cdnsciencepub.com/authors-and-reviewers/writing-a-plain-language-summary> | 2022 | EU-CTR characteristics, LS content, Reading tool, LS number of words |
|  | Grey literature | Center for community engagement and impact, The Power of Translating your Research: How to Write an Effective Lay Abstract and Impact Statement, <https://research.vcu.edu/media/office-of-research-and-innovation/humanresearch/power-of-translating.pdf> | 2021 | EU-CTR characteristics, LS content, Reading tool, Reading level, |
|  | Grey literature | Children’s Cancer and Leukemia Group, Writing a LS, <https://www.tyac.org.uk/downloads/research/cclg-writing-a-lay-summary-guidance-july-2017.pdf> | 2017 | EU-CTR characteristics, LS content, Reading tool, LS number of words |
|  | Grey literature | Cochrane Methods, Standards for reporting Plain Language Summaries in New Cochrane Intervention Reviews-2013, <https://consumers.cochrane.org/sites/consumers.cochrane.org/files/uploads/pleacs_2019.pdf> | 2013 | EU-CTR characteristics, LS content, Reading tool, Reading level LS writing guidance, LS number of words |
|  | Grey literature | Cochrane Norway1, How to write a plain language summary of a Cochrane intervention review- 2019. <https://www.cochrane.no/sites/cochrane.no/files/uploads/how_to_write_a_cochrane_pls_12th_february_2019> | 2019 | EU-CTR characteristics, LS content, |
|  | Grey literature | Cochrane collaboration-Standards, Standards for reporting Plain Language Summaries (PLS) for Cochrane Diagnostic Test Accuracy Reviews-2014. <http://methods.cochrane.org/sites/methods.cochrane.org.sdt/files/uploads/Draft%20PLS%20document.pdf> | 2014 | EU-CTR characteristics, LS content, Reading tool, Reading level, LS writing guidance |
|  | Grey literature | Cochrane, Template and guidance for writing a Cochrane Plain language summary-2022, <https://training.cochrane.org/guidance-writing-cochrane-plain-language-summary.pdf> | 2022 | EU-CTR characteristics, LS content, Reading tool, Reading level, |
|  | Grey literature | Cochrane Norway 2, How to write a plain language summary of a Cochrane intervention review-checklist-2016, <https://www.cochrane.no/sites/cochrane.no/files/uploads/checking_a_cochrane_pls_15th_june_2018.pdf> | 2016 | LS content, LS number of words, EU-CTR characteristics |
|  | Grey literature | Clinical Trials Expert Group, Good LS Practice, <https://health.ec.europa.eu/system/files/2021-10/glsp_en_0.pdf> | 2021 | EU-CTR characteristics, LS content, Reading tool, Reading level, |
|  | Grey literature | Clinical Trial Ontario, Plain Language Result Summaries- adult clinical trials, <https://www.ctontario.ca/patients-public/resources-for-engaging-patients/toolkit-to-improve-clinical-trial-participants-experiences/plain-language-summaries/> | 2020 | EU-CTR characteristics, |
|  | Grey literature | Clinical Trial Ontario-Paediatric, Plain Language Result Summary- Paediatric Clinical Trials, <https://www.ctontario.ca/cms/wp-content/uploads/2022/05/CommuniKIDS-Considerations-Tipsheet-May-2022-Final.pdf> | 2022 | EU-CTR characteristics, |
|  | Grey literature | Developmental Disabilities Network Journal, Editorial Guidelines & Submission Policies, <https://digitalcommons.usu.edu/ddnj/policies.html> | 2022 | EU-CTR characteristics, LS number of words, Reading level |
|  | Grey literature | Digital Curation Centre, How To Write a LS, <https://www.dcc.ac.uk/sites/default/files/documents/publications/HowToLaySummariesDec2012.pdf> | 2012 | EU-CTR characteristics, LS content, |
|  | Grey literature | eLife, Plain-language Summaries: How to write an eLife digest, <https://elifesciences.org/inside-elife/85518309/plain-language-summaries-how-to-write-an-elife-digest> | 2017 | EU-CTR characteristics, LS number of words |
|  | Grey literature | eLife, Plain-language Summaries: Results of the 2016 eLife digest reader survey, <https://elifesciences.org/inside-elife/19c97b89/plain-language-summaries-results-of-the-2016-elife-digest-reader-survey> | 2017 | EU-CTR characteristics, LS content,, LS number of words |
|  | Grey literature | Elsevier, In a nutshell: how to write a LS <https://www.elsevier.com/connect/authors-update/in-a-nutshell-how-to-write-a-lay-summary> | 2018 | EU-CTR characteristics, LS content, |
|  | Grey literature | Envision Pharma Group. Plain Language Summaries (PLS) of Publications Toolkit, <https://www.envisionthepatient.com/plstoolkit> | 2022 | EU-CTR characteristics, LS content, |
|  | Grey literature | European Federation of Pharmaceutical Industries and Associations, Reflection Paper- EFPIA Guiding Principles on Layperson Summary, <https://www.efpia.eu/media/25661/reflection-paper-efpia-guiding-principles-on-layperson-summary.pdf> | 2014 | EU-CTR characteristics, LS content, Reading level |
|  | Grey literature | European Patient Forum, EPF position: Clinical trial results –  communication of the LS, <https://www.eu-patient.eu/globalassets/policy/clinicaltrials/epf-lay-summary-position-final_external.pdf> | 2015 | EU-CTR characteristics, LS content, |
|  | Grey literature | Evidence for Democracy, Preparing Plain Language Summaries, <https://drive.google.com/file/d/1d-NSSG-kd-vysFBB5vJUmCreAILGGrbX/view> | 2021 | EU-CTR characteristics, LS content, Reading tool, LS number of words |
|  | Grey literature | FACETS, Instructions to Authors Journal, <https://www.facetsjournal.com/for-authors/instructions-to-authors> | 2022 | EU-CTR characteristics, LS content, LS number of words |
|  | Grey literature | Future Medicine, Plain Language Summaries. <https://www.futuremedicine.com/plainlanguagesummaries> | 2022 | EU-CTR characteristics,, LS number of words |
|  | Grey literature | Journal of Cystic Fibrosis, Cystic Fibrosis Research News, <https://www.cysticfibrosisjournal.com/content/authorinfo> | 2022 | EU-CTR characteristics, LS content,, LS number of words |
|  | Grey literature | Indian Journal of dermatology and venereology and leprology, Instructions for Authors, <https://ijdvl.com/contributors/> | 2022 | EU-CTR characteristics, LS number of words, Reading level |
|  | Grey literature | International Alliance of Academic childhood disability, Translating a Publication to a Lay Language Summary, <https://iaacd.net/2021/04/22/translating-a-publication-to-a-lay-language-summary/> | 2018 | EU-CTR characteristics, LS content, LS development process, Reading level |
|  | Grey literature | Iranian Rehabilitation Journal-Author instruction, Authors Instructions, <http://irj.uswr.ac.ir/page/21/Authors-Instruction> | 2022 | EU-CTR characteristics, LS content,, LS number of words |
|  | Grey literature | iTech post, Future Science Group Explains How to Write Plain Language Summaries, <https://www.itechpost.com/articles/108569/20220104/future-science-group-explains-how-to-write-plain-language-summaries.htm> | 2022 | EU-CTR characteristics, Reading tool, Reading level, LS development process, |
|  | Grey literature | Kidney Foundation, Best Practices for Writing a Plain Language Summary, <https://kidney.ca/getattachment/Research/Funding-Opportunities/Accordion/Allied-Health-Kidney-Scholarships/Best-Practices-FINAL-English.pdf?lang=en-CA> | 2018 | EU-CTR characteristics, LS content, |
|  | Grey literature | Lionbridge, Successfully Authoring and Translating Plain Language Summaries, <http://docplayer.net/180610890-Successfully-authoring-and-translating-plain-language-summaries.html> | 2019 | EU-CTR characteristics, Reading tool, Reading level, |
|  | Grey literature | Medical Writing Journal, The ABCs of pediatric plain language summaries, <https://journal.emwa.org/mentorship/the-abcs-of-paediatric-plain-language-summaries/> | 2021 | EU-CTR characteristics, Reading level, |
|  | Grey literature | Medical affairs professional society, The role of PLS in communicating clinical trial Data, <https://medicalaffairs.org/wp-content/uploads/2022/05/MAPS-Elevate-TheRoleOfPlainLang-V2.pdf> | 2022 | EU-CTR characteristics, |
|  | Grey literature | Michael Smith Health Research BC, <https://healthresearchbc.ca/bc-support-unit/info-and-resources/information-for-researchers/plain-language-guide/> | 2018 | EU-CTR characteristics, Reading tool, Reading level, |
|  | Grey literature | National Health Service, Writing a plain language (lay) summary of your research findings, <https://www.hra.nhs.uk/planning-and-improving-research/best-practice/writing-plain-language-lay-summary-your-research-findings/> | 2021 | EU-CTR characteristics, LS content,, LS number of words |
|  | Grey literature | National Institute for Health and Care Research, Plain English summaries, <https://www.nihr.ac.uk/documents/plain-english-summaries/27363> | 2021 | EU-CTR characteristics, LS content, |
|  | Grey literature | Ontario HIV Treatment Network , Plain Language Summary Guidelines, <https://www.ohtn.on.ca/plain-language-2/> | 2022 | EU-CTR characteristics, LS content,, LS number of words |
|  | Grey literature | Patient focus medical development, Plain language summaries (PLS) of peer-reviewed publications and conference presentations: Practical "How-To" Guide for multi-stakeholder co-creation, <https://pemsuite.org/How-to-Guides/WG5.pdf> | 2022 | EU-CTR characteristics, LS content, Reading tool, LS development process, |
|  | Grey literature | People and Nature Journal, Plain Language Summary Guidelines, <https://besjournals.onlinelibrary.wiley.com/pb-assets/hub-assets/besjournals/2575314_PaN/People%20and%20Nature%20Plain%20Language%20Summary%20Guidelines-1540981909900.pdf> | 2022 | EU-CTR characteristics, LS number of words |
|  | Grey literature | Perfect it, Hacks to Nail Your Next Plain Language Summary, <https://intelligentediting.com/blog/8-hacks-to-nail-your-next-plain-language-summary/> | 2021 | EU-CTR characteristics, LS content, Reading tool, Reading level, |
|  | Grey literature | Plain Language Summaries, Frequently Asked Questions  Everything you need to know about publishing a plain language summary, <https://www.plainlanguagesummaries.com/plain-language-summaries-faq/> | 2022 | EU-CTR characteristics, |
|  | Grey literature | SAGE Publishing, Plain Language Summaries, <https://languageservices.sagepub.com/en/files/plain-language-summaries-external-guidelines.pdf> | 2022 | EU-CTR characteristics, LS content,, LS number of words |
|  | Grey literature | Science News by AGU (Advancing Earth and Space Science), Plain Language Summaries Explained in Plain Language, <https://eos.org/editors-vox/plain-language-summaries-explained-in-plain-language> | 2021 | EU-CTR characteristics, LS content, LS number of words, Reading level |
|  | Grey literature | Springer HealthCare, Guidelines for digital features and plain language summaries, <https://springerhealthcare.com/wp-content/uploads/2021/08/Guidelines-for-digital-features-and-plain-language-summaries-v1.1.pdf> | 2021 | EU-CTR characteristics,, LS number of words |
|  | Grey literature | The Academy of Medical Science, Top tips for writing a LS, <https://acmedsci.ac.uk/more/news/10-tips-for-writing-a-lay-summary> | 2014 | EU-CTR characteristics, LS content, |
|  | Grey literature | TransCelerate Biopharma Inc 2, Recommendations For Drafting Non- Promotional Lay Summaries of Clinical Trial Results, <http://www.transceleratebiopharmainc.com/wp-content/uploads/2015/04/TransCelerate-Non-Promotional-6Language-Guidelines-v10-1.pdf> | 2015 | EU-CTR characteristics, |
|  | Grey literature | TransPerfect, Plain-Language Abstracts: A Benefit to Your Research, <https://www.transperfect.com/blog/plain-language-abstracts-benefit-your-research> | 2020 | EU-CTR characteristics, LS content, Reading level |
|  | Grey literature | Trilogy writing and consulting, The LS – Remember the Reader, <https://www.trilogywriting.com/wp-content/uploads/2017/12/39_The-Lay-Summary-Remember-the-Reader_Lisa-Chamberlain-James-and-Barry-Drees_Journal-for-Clinical-Studies-9-6_Dec-2017.pdf> | 2017 | EU-CTR characteristics, LS content, |
|  | Grey literature | Tylor and Francis, How to write a Plain Language Summary, <https://authorservices.taylorandfrancis.com/wp-content/uploads/2022/03/TF-PLS-Guidelines-for-Authors-UPDATE-10-Mar-22-FINAL.pdf> | 2022 | EU-CTR characteristics, LS content, |
|  | Grey literature | UK Alzheimer, Writing a good LS of your research, <https://www.alzheimersresearchuk.org/wp-content/uploads/2015/03/Writing-a-good-lay-summary-of-your-research-2020.pdf> | 2020 | EU-CTR characteristics, LS content, |
|  | Grey literature | University of Oxford-Communication Hub, <https://communications.admin.ox.ac.uk/resources/how-to-guide-writing-a-lay-summary> | 2022 | EU-CTR characteristics, LS content, |
|  | Grey literature | U.S. Department of Education, Going public: Writing about research in everyday language, <https://ies.ed.gov/ncee/pubs/REL2014051/pdf/REL_2014051.pdf> | 2014 | EU-CTR characteristics, |
|  | Grey literature | Wiley, How to Write A LS for Your Research, <https://www.wiley.com/network/societyleaders/research-impact/how-to-write-a-lay-summary-for-your-research> | 2019 | EU-CTR characteristics, LS content, LS number of words |
|  | Peer reviewed | Anstey et al., Plain language summaries in the British Journal of Dermatology: connecting with patients, Br J Dermatol. 2014 Jan;170(1):1-3. | 2014 | EU-CTR characteristics, LS number of words |
|  | Peer reviewed | Barnes et al., Lay Summaries of Clinical Study Results: An Overview. Pharmaceut Med. 2019 Aug;33(4):261-268. | 2019 |  |
|  | Peer reviewed | Barnfield et al., Is all the stuff about neurons necessary?” The development of lay summaries to disseminate findings from the Newcastle Cognitive Function after Stroke (COGFAST) study. Res Involv Engagem. 2017; 3: 18. | 2017 | EU-CTR characteristics, LS content, Reading tool, LS development process, |
|  | Peer reviewed | Dormer et al., A practical ‘How-To’ Guide to plain language summaries (PLS) of peer-reviewed scientific publications: results of a multi-stakeholder initiative utilizing co-creation methodology. Res Involv Engagem. 2022.8, 23 | 2022 | EU-CTR characteristics, Reading tool, LS development process, |
|  | Peer reviewed | Dubé et al., Lay Abstracts and Summaries: Writing Advice for Scientist. J Cancer Educ. 2014 Sep;29(3):577-9. | 2014 | EU-CTR characteristics, Reading tool, Reading level, LS development process, |
|  | Peer reviewed | Duke et al., Lay Summaries for Research Articles: A Citizen Science Approach to Bridge the Gap in Access. ELPUB. 2015: 1-7 | 2015 | EU-CTR characteristics, |
|  | Peer reviewed | James et al., Lay summaries and writing for patients: Where are we now and  where are we going? Trends in medical writing. 2019; 28(3) | 2019 | EU-CTR characteristics, |
|  | Peer reviewed | Kornør et al., Systematic reviews on child welfare services: identifying and disseminating the evidence. J Eval Clin Pract. 2015 Oct;21(5):855-60. | 2015 | EU-CTR characteristics, LS development process, LS content |
|  | Peer reviewed | Maurer et al., Lessons Learned from Developing Plain Language Summaries of Research Studies. Health Lit Res Pract. 2021 Apr;5(2):e155-e161. | 2021 | EU-CTR characteristics, LS content, Reading tool, Reading level, LS development process, |
|  | Peer reviewed | Mogami et al., Creation of a patient-centric patient LS in the local language. Medical Writing. 2017; 26 (4). | 2017 | EU-CTR characteristics, LS development process, Reading level |
|  | Peer reviewed | Rosenberg b (openpharma) et al., Open Pharma recommendations for plain  language summaries of peer-reviewed medical journal publications | 2021 | EU-CTR characteristics, LS number of words |
|  | Peer reviewed | Salita et al., Writing for lay audiences: A challenge for scientists. Curr Med Res Opin. 2021 Nov;37(11):2015-2016. | 2015 | EU-CTR characteristics, LS content, Reading tool, |
|  | Peer reviewed | Sedgwick et al., Extending the reach of science – Talk in plain language. Epilepsy Behav Rep. 2021 Oct 25; 16:100493. | 2021 | EU-CTR characteristics, Reading tool, |
|  | Peer reviewed | Wada M et al., A protocol for co-creating research project lay summaries with stakeholders: guideline development for Canada’s AGE-WELL network. Res Involv Engagem. 2020 May 8; 6:22. | 2020 | EU-CTR characteristics, LS content, LS development process, |
|  | Peer reviewed | White et al., Developing Lay Summaries as a Bidirectional Learning Opportunity for Authors and Undergraduate Scholars: The Jackson Heart Study. Pedagogy in Health Promotion. 2020; 6(2):237337991984225 | 2020 | EU-CTR characteristics, LS content, Reading tool, Reading level, |

*LS: Lay Summary
